# Health education program improves QOL in students with irritable bowel syndrome after the Wenchuan earthquake: a five-year multi-center study

**DOI:** 10.1186/s12876-018-0845-4

**Published:** 2018-07-27

**Authors:** Shi-Cheng Zheng, Hui Gong, Yi-Ping Wang, Qiang Zhang, Li-Li Wang, Xue-Fen Liao, Dai-Wen He, Jing Wu

**Affiliations:** 1 Department of Gastroenterology, The First People’s Hospital of Longquanyi District, Chengdu & West China Longquan Hospital Sichuan University, Sichuan Province, Chengdu, 610100 China; 2 Department of Gastroenterology, West China Hospital, Sichuan University, Sichuan Province, Chengdu, 610041 China; 3 West China School of Public Health, Sichuan University, Chengdu, 610041 Sichuan Province China; 4 Qingchuan Middle School, Qingchuan County, 628100 Sichuan Province China; 5 Wenchuan First Middle School, Wenchuan County, 638100 Sichuan Province China; 6 Tongji Middle School, Pengzhou City, 611900 Sichuan Province China; 7 Longquan District of Chengdu Maternity and Child Health Care Hospital, Sichuan Province, Chengdu, 610100 China

**Keywords:** Health education, Irritable bowel syndrome, Middle school students, The centralized health education program, Quality of life, Earthquake

## Abstract

**Background:**

Stress is a common contributing factor for irritable bowel syndrome (IBS). This study was to evaluate the efficacy of the centralized health education program in improving the quality of life (QOL) of middle school students with IBS who experienced the Wenchuan earthquake on May 12, 2008.

**Methods:**

A multi-center, randomized and open evaluation study design was adopted. A total of 584 students who met the Rome III criteria for IBS in four middle schools were identified. Of these students, 29 were excluded for various reasons, and the remaining 555 students were randomly assigned to either the health education group (*n* = 277) or the control group (*n* = 278, received no health education). De-identified data were collected via the IBS quality of life (IBS-QOL) questionnaire and abdominal pain was assessed during the 5-year follow-up survey.

**Results:**

The IBS-QOL mean total score was comparable at baseline between no-education group and education group no matter in quake-unaffected areas or quake-affected areas (52.27 vs 51.43, *t* = 1.15, *P* > 0.05; 51.02 vs 50.64, *t* = 1.98, *P* > 0.05). During the 5-year study period, 84 students opted out during follow-up. After 5 years, a significant difference of the IBS-QOL mean total score was observed between the no-education group and education group in quake-unaffected areas (80.53 vs 93.67, *t* = − 55.45, *P* < 0.01), which was also observed in quake-affected areas (64.23 vs 93.80, *t* = − 188.10, *P* < 0.01). In addition, there was a reciprocal action between factor 1(health education or not) and factor 2(affected by the earthquake or not) regarding IBS-QOL for dysphoria(Q1), interference with activity(Q2), food avoidance(Q5) and relationships(Q8)(*P* < 0.001) at year 1, 3 and 5. In all students, abdominal pain scores gradually reduced from baseline in each subgroup over 5 years (*P* < 0.001).The improvement was greater in the education group than in the control group no matter in quake-unaffected area and in quake-affected areas(*P* < 0.001). There was a reciprocal action between factor 1(health education or not) and factor 2(duration of follow-up) regarding the mean abdominal pain symptom score irrespective of quake-unaffected or quake-affected areas (*P* = 0.029 and *P* < 0.001).

**Conclusion:**

The health education program improved quality of life and abdominal pain in middle school IBS students in Wenchuan quake-affected areas.

## Background

Irritable bowel syndrome (IBS) is a set of functional gastrointestinal diseases lacking morphological or abnormal biochemical indicators, which mainly manifests as abdominal pain, distention or discomfort with changes in bowel habit and abnormal stool characteristics. Most patients according to the Rome III diagnostic criteria,but the causes of the disease are not yet clear and symptoms recurrent [1–3].Some of the studies have focused on anxiety, depression and intestinal microecological changes [4], various treatments and drugs have been used in IBS patients, but with limited efficacy [5, 6]. Therefore, the Rome foundation has proposed a multidimensional clinical data analysis method, which integrated the symptoms experienced by IBS patients (including clinical symptoms, social psychology, physiology, life quality, etc) and the IBS patient’s individualized treatment plan. Since our earlier research has found that earthquakes may cause or aggravate the onset of IBS in middle school students, so we aimed to implement the centralized health education program to reveal its role in the treatment of IBS. Many studies have shown that health education program play a vital role in the treatment of IBS, and IBS-specific quality of life (IBS-QOL) can be used as a treatment evaluation tool [7–9]. Boersma et al [10] showed in a single-case experimental design in 13 subjects that cognitive behavioral therapy (CBT) for IBS can significantly reduce gastrointestinal symptoms, pain catastrophizing and QOL. Liegl et al [11] believe that guided self-help (GSH) is not only effective but easy to implement. We have demonstrated that the incidence rate of anxiety-related emotional disorders among students suffering from IBS who experienced the Wenchuan earthquake on May 12, 2008, was higher than that among non-IBS students and students unaffected by the earthquake [12]. However, there are no reports on whether health education programs have an impact on IBS among those who experienced an earthquake.

## Methods

### Study setting and participants

**Fig. 1:**
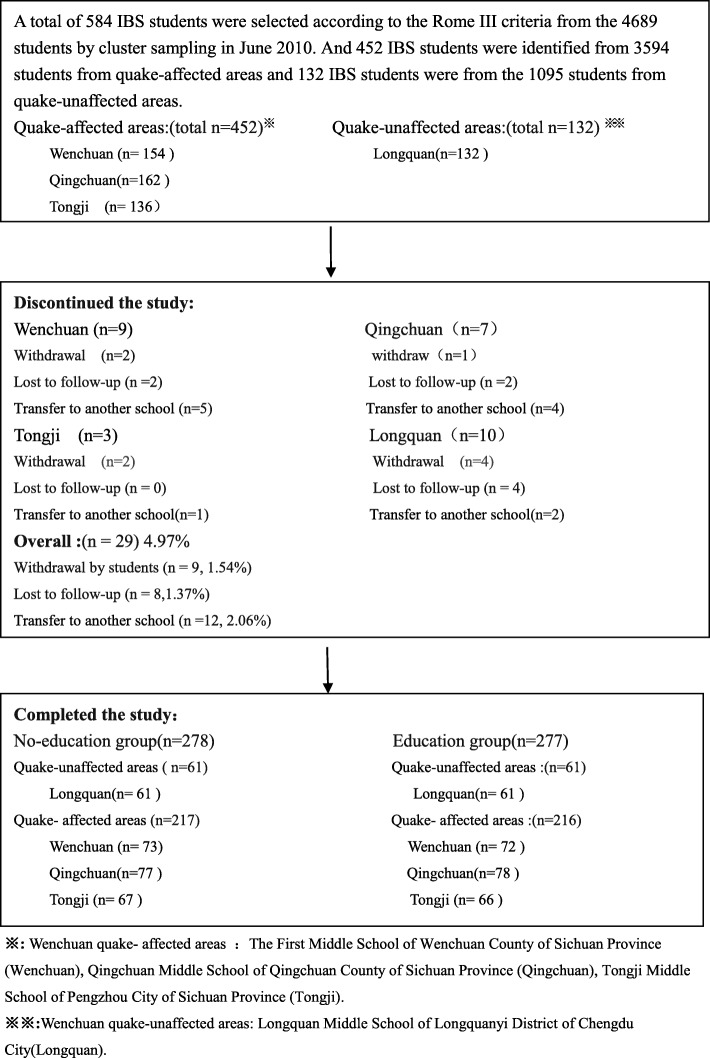
Composition of the enrolled students. **※** Wenchuan quake-affected areas: The First Middle School of wenchuan County of Sichuan Province (Wenchuan), Qingchuan Middle School,of Qingchuan County of Sichuan Province (Qingchuan), Tongji Middle School of Pengzhou City of Sichuan Province (Tongji). **※※**Wenchuan quake-unaffected areas:Longquan Middie School of Longquanyi Districtof Chengdu City(Longquan)

The study was conducted from June 2010 to December 2015 in three middle schools(Qingchuan Middle School, Wenchuan No. 1 Middle School and Tongji Middle School) in the quake-affected areas (Wenchuan on May 12, 2008) and Longquan Middle School in the quake-unaffected area. A total of 584 IBS students were selected from 3594 students in quake-affected areas and 1095 students in quake-unaffected areas according to the Rome III criteria by cluster sampling in June 2010(The Wenchuan earthquake occurred two years ago). Twenty-nine of these 584 students were excluded from the final analysis due to: withdrawal (*n* = 9); lost to follow-up (*n* = 8); transfer to other schools not in the study area (*n* = 12). Thus, a total of 555 IBS students were assigned to the health education group (*n* = 277) and the control group (*n* = 278) using cluster random sampling method according to the class number (Fig. 1). All IBS students who were assigned to the health education group received a multi-center health education program.

### Health education program

#### Program of health education

We designed this study for middle school IBS students in the 7th to 9th grade in Wenchuan earthquake areas, whose symptoms met those of the Rome III criteria. In the Chinese education system, the 9th grade is still part of middle school. School doctors or psychology teachers were selected to receive our standard IBS training. They provided health education to these students collectively, combined with individual guidance if needed. The IBS students were evaluated twice a month during the first 2 years, and once a month in the subsequent 3 years.

The IBS health education program [13] contained the following: 1. what does it mean to be diagnosed with IBS; 2. causes and manifestations of IBS, such as abdominal pain, diarrhea, constipation, abdominal distention and extra-gastrointestinal presentations; 3. ways to relieve stress (as effective treatment of stress is an important measure in reducing IBS symptoms); 4. benefits and methods of physical exercise, and an explanation that those who exercise less need to gradually increase the intensity /duration; and 5. regulation of eating habits: an explanation that some IBS symptoms may be aggravated by food; to ensure that the students will not fear eating but understand that foods affect individuals differently; guidance on food and drink choices that may cause abdominal pain, diarrhea, flatulence and constipation.

#### Evaluation program

Students completed the IBS—QOL special measurement form at the beginning of the health education program (2010), in the first year (2011), third year (2013) and fifth year (2015). If students did not understand the contents, the school doctor or psychology teacher would clarify. However, the final answers in the survey were made solely by the student. The school doctor or psychology teacher checked whether there were any unanswered questions when the forms were collected to ensure the completeness of the survey. Those who were not willing to participate or could not continue the health education program were excluded.

### IBS—QOL scale [14–16]

The IBS—QOL Scale prepared by Patrick et al. was adopted and was translated into Chinese.After carrying out cultural proofreading and validity test,the IBS—QOL Scale is suitable for Chinese [17].

It was composed of 34 items reflecting 8 fields including dysphoria (Q1), interference with activity (Q2), body image (Q3), health worries (Q4) food avoidance (Q5), social reactions (Q6), sexual concerns (Q7) and relationships (Q8), of which the item on “sexual intercourse” was deleted as it was not applicable to these middle school students (according to the Chinese National Condition). Therefore, there was a total of 32 items in the survey reflecting 7 fields. Each item was scored according to the method of Likert: 1 = not at all, 2 = slightly, 3 = moderately, 4 = quite a bit, 5 = completely. The scores were calculated using the following formula: {1-[(A-B)÷(C-B)]} × 100 (A = the total value of the item;B = the lowest score theory;C = the theory of the highest score). The transformed scores ranged from 0 to 100, with higher values indicating a better quality of life.

#### Abdominal pain symptom scores

We used the following Visual Analogue Scale/Score (VAS) to detect the degree of abdominal pain: “0” represented no distress of abdominal pain; “1–2”, annoying pain; “2–4”, uncomfortable pain; “4–6”,dreadful pain; “6–8”, horrible pain; “8–9”, agonizing pain; and “10”, unbearable abdominal pain.

### Statistical methods

SAS9.0 statistical software was used to generate random numbers and analyze the data. The IBS—QOL and VAS measure were taken as the evaluation standard of the influence of QOL and abdominal pain before and after the education program. EpiData 3.1 was used to record the data in the IBS—QOL Scale and abdominal pain scores, which were evaluated by SPSS17.0 statistical analysis. Comparisons between categorical data were conducted using the Chi-square test, and the scoring data were evaluated by the student’s t test between education group and no-education group, and by the factorial design analysis of variance in different years between education group and no-education group, with *P* = 0.05 as the cutoff.

## Results

### Student demographics

**Fig. 2:**
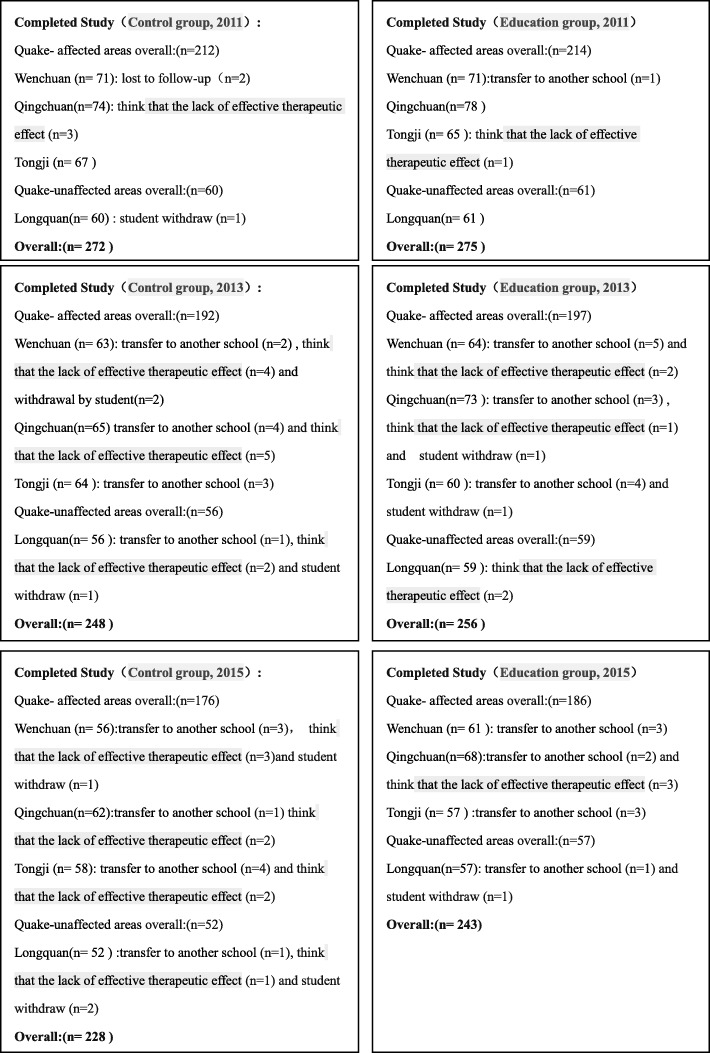
Composition of the enrolled students during the 5-year follow-up

In the end, 555 middle school students who were diagnosed with IBS were enrolled in the study for analysis, of which 277 students were assigned to the health education group and 278 students were assigned to the control group using cluster random sampling method (Fig. 1). During the 5-year study period, 84 students opted out during follow-up (Fig. 2). In Longquanyi District(quake-unaffected area), the average age of the students from the no-education group(control group) was significantly younger than that from the education group(13.8 ± 1.65 vs 15.1 ± 1.75, *P* < 0.001). Whereas, the average age of the students from the no-education group was significantly older than that from the education group(14.4 ± 1.49 vs 13.8 ± 1.40, *P* < 0.05) in Tongji Town(one of the quake-affected areas). In Tongji Town, the difference in the gender composition was significant statistically (*P* = 0.01) between the no-education group and the education group, as 42 students were female (42/67,62.69%) in the former group, whereas 27 students were female (27/66,40.91%) in the latter group.

Moreover, no significant difference was found in the ethnicity composition, subgroups of IBS between the education group and no-education group in each area (Table 1 and Table 2).

**Table 1:** Demographics of the students from middle schools in various areas

Characteristics(unit)	No-education group Mean(SD) or *n*(%)	Education group Mean(SD) or *n*(%)	*Statistics*	*P*
Longquanyi District				
Age(years)	13.8(1.65)	15.1(1.75)	−4.22	0.001**
Gender				
Male	25(40.98)	21(34.43)	0.56	0.45
Female	36(59.02)	40(65.57)		
Ethnicity				
Han	61(100.00)	61(100.00)	–	1.00*
Tibetan	0(0.00)	0(0.00)		
Qiang	0(0.00)	0(0.00)		
Hui	0(0.00)	0(0.00)		
Wenchuan County				
Age(years)	15.2(1.64)	15.5(1.69)	−1.22	0.23
Gender				
Male	36(49.32)	34(47.22)	0.04	0.85
Female	37(50.68)	38(52.78)		
Ethnicity				
Han	42(57.53)	35(48.61)	–	0.20*
Tibetan	4(5.48)	7(9.72)		
Qiang	27(36.99)	29(40.28)		
Hui	0(0.00)	1(1.39)		
Qingchuan County				
Age(years)	15.7(1.41)	15.7(1.39)	0.38	0.70
Gender				
Male	30(38.96)	33(42.31)	0.74	0.39
Female	47(60.14)	45(57.69)		
Ethnicity				
Han	61(79.22)	68(87.18)	1.16	0.28
Tibetan	0(0.00)	0(0.00)		
Qiang	0(0.00)	0(0.00)		
Hui	16(20.78)	10(12.82)		
Tongji Town				
Age(years)	14.4(1.49)	13.8(1.40)	2.22	0.03**
Gender				
Male	25(37.31)	39(59.09)	6.31	0.01**
Female	42(62.69)	27(40.91)		
Ethnicity				
Han	67(100.00)	66(100.00)	–	1.00*
Tibetan	0(0.00)	0(0.00)		
Qiang	0(0.00)	0(0.00)		
Hui	0(0.00)	0(0.00)		

*Fisher’s test. SD, standard deviation.**represents that the difference is statistically significant

**Table 2:** Subgroups of IBS students from middle schools in Longquanyi, Wenchuan, Qingchuan and Tongji

Characteristics	No-education group, *n*(%)	Education group, *n*(%)	*Statistics*	*P*
Longquanyi District				
IBS-C	21(34.43)	15(24.59)	–	0.25*
IBS-D	31(50.82)	34(55.74)		
IBS-M	7(11.48)	5(8.20)		
Unknown	2(3.28)	7(11.48)		
Wenchuan County				
IBS-C	18(24.66)	21(29.17)	0.95	0.81
IBS-D	42(57.53)	38(52.78)		
IBS-M	9(12.33)	7(9.72)		
Unknown	4(5.48)	6(8.33)		
Qingchuan County				
IBS-C	22(28.57)	20(25.64)	2.20	0.53
IBS-D	47(61.04)	41(52.56)		
IBS-M	5(6.49)	10(12.82)		
Unknown	3(3.90)	7(8.97)		
Tongji Town				
IBS-C	18(26.87)	19(28.79)	0.84	0.84
IBS-D	39(58.21)	34(51.52)		
IBS-M	6(8.96)	7(10.61)		
Unknown	4(5.97)	6(9.09)		

*fisher’s test, IBS-C: IBS with constipation, IBS-D: IBS with diarrhoea, IBS-M: IBS with mixed type, IBS-U: IBS with uncertainty

### IBS subgroups

IBS with diarrhea (IBS-D) was more prevalent than IBS with constipation(IBS-C), IBS with mixed type (IBS-M) and IBS with uncertainty (IBS-U) both in the quake-unaffected areas (Longquanyi District) and quake-affected areas (Wenchuan County, Qingchuan County and Tongji Town). However, there were no significant differences in the ratio of IBS subgroups between the non-education group and education group in these four areas (*P* > 0.05) (Table 2).

### Primary outcomes

Table 3 showed the changes of IBS-QOL total score from baseline in 5 years and lists the mean value of IBS-QOL total score at baseline (2010), after 1 year (2011), 3 years (2013) and 5 years(2015) between the no-education and education group. A comparison of the no-education group and education groups showed that the IBS-QOL mean total score was comparable at baseline (51.28 vs 50.81, *t* = 2.15, *P* > 0.05). After 5 years, the difference between the two groups was significant (67.61 vs 93.77, *t* = − 64.03, *P* < 0.01). A comparison between baseline and year 5(2015) in the no-education group displayed that the IBS-QOL mean total score increased dramatically(51.28 vs 67.61,*t* = − 34.93, *P* < 0.001), which was also observed in the education group(50.81 vs 93.77, *t* = − 330.4, *P*<0.001).

**Table 3:** Changes of IBS-QOL total score from baseline in 5 years between the no-education and education group

	No-education group			Education group			*t*	*P*
	*n*	Mean	SD	*n*	Mean	*S*D		
QOL Total Score(2010)	278	51.28	3.32	277	50.81	1.62	2.15	> 0.05
QOL Total Score(2011)	272	62.49	2.87	275	74.87	3.54	−45.72	< 0.01*
QOL Total Score(2013)	248	66.67	4.18	256	85.15	1.82	−69.21	< 0.01*
QOL Total Score(2015)	228	67.61	6.88	243	93.77	1.30	−64.03	< 0.01*

*represents that the difference is statistically significant

Furthermore, it also showed that the IBS-QOL mean total score was comparable at baseline between no-education group and education group no matter in quake-unaffected areas or quake-affected areas (52.27 vs 51.43, *t* = 1.15, *P* > 0.05; 51.02 vs 50.64, *t* = 1.98, *P* > 0.05) from Table 4. After 5 years, the difference between the two groups was significant no matter in quake-unaffected areas or quake-affected areas (80.53 vs 93.67, *t* = − 55.45, *P* < 0.01; 64.23 vs 93.80,*t* = − 188.10, *P* < 0.01). In quake-unaffected areas, a comparison between baseline and year 5(2015) in the no-education group displayed that the IBS-QOL mean total score increased dramatically(52.27 vs 80.53, *t* = − 35.70, *P* < 0.001), which was also observed in the education group (51.43 vs 93.67, *t* = − 182.7, *P* < 0.001). In quake-affected areas, a comparison between baseline and year 5(2015) in the no-education group displayed that the IBS-QOL mean total score also increased dramatically(51.02 vs 64.23, *t* = − 59.30, *P*<0.001), which was also observed in the education group (50.64 vs 93.80, *t* = − 284.6, *P*<0.001).

**Table 4:** Changes in IBS-QOL total score from baseline in quake-unaffected areas or quake-affected areas and no-education or education group at years of 2010, 2011,2013 and 2015

	No-education group			Education group			*t*	*P*
	*n*	Mean	*SD*	*n*	Mean	*SD*		
Quake-unaffected areas								
IBS-QOL Total Score(2010)	61	52.27	5.55	61	51.43	1.33	1.15	0.26
IBS-QOL Total Score(2011)	60	62.06	3.11	61	74.12	1.79	−26.27	< 0.01*
IBS-QOL Total Score(2013)	56	73.62	1.63	59	84.80	1.26	−42.38	< 0.01*
IBS-QOL Total Score(2015)	52	80.53	1.43	57	93.67	1.17	−55.45	< 0.01*
Quake-affected areas								
IBS-QOL Total Score(2010)	217	51.02	2.37	216	50.64	1.66	1.98	> 0.05
IBS-QOL Total Score(2011)	212	62.60	2.80	214	75.08	3.87	−38.85	< 0.01*
IBS-QOL Total Score(2013)	192	64.85	2.30	197	85.23	1.94	−101.76	< 0.01*
IBS-QOL Total Score(2015)	176	64.23	1.96	186	93.80	1.33	−188.10	< 0.01*

*represents that the difference is statistically significant

**Fig. 3:**
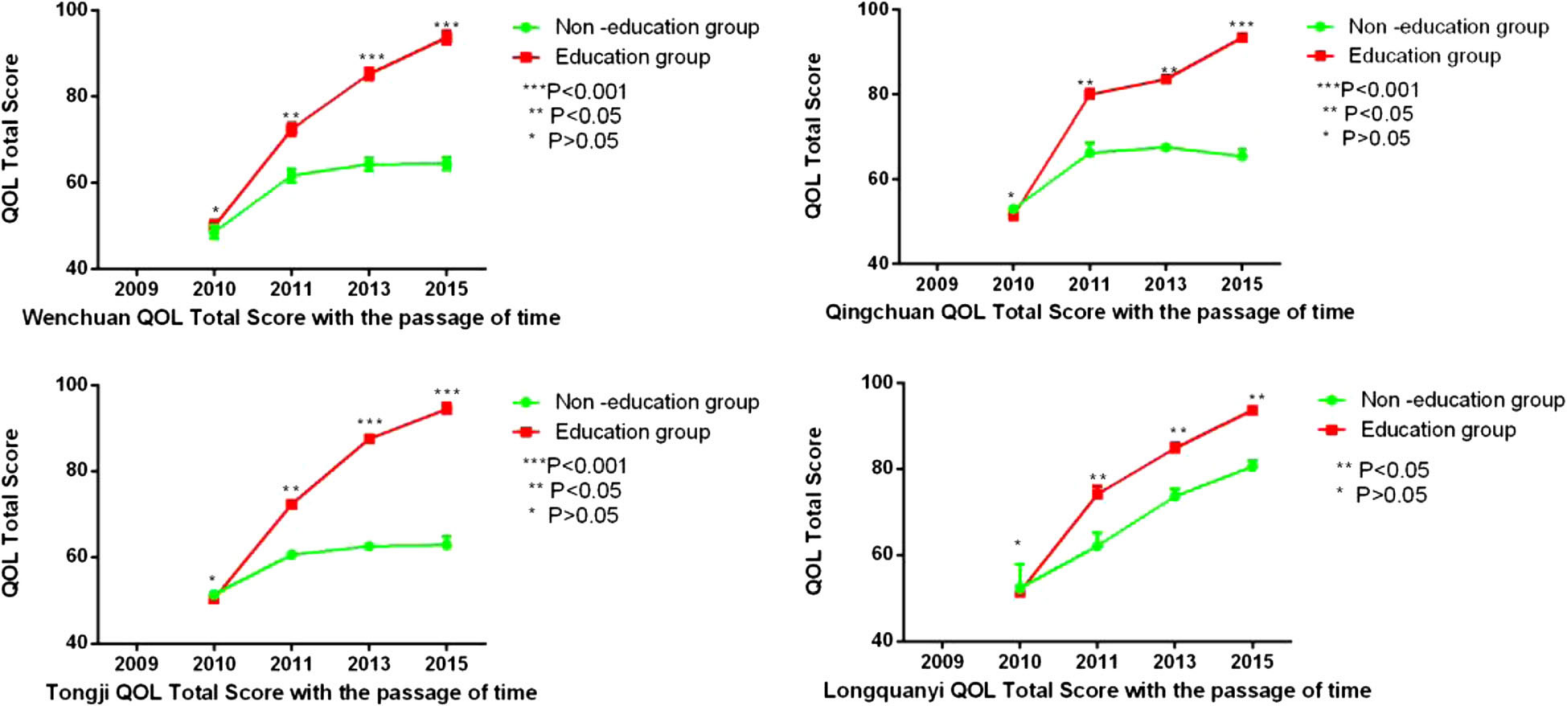
The increase in IBS-QOL total score from baseline in the education group and no-education group in each school at year of 2010;2011;2013;2015. ^**^; ^***^represent that the difference is statistically significant

Figure 3 also showed that the IBS-QOL mean total score was comparable at baseline between no-education group and education group no matter in Longquanyi(the only one school from quake-unaffected areas) or in the other three schools(Wenchuan, Qingchuan and Tongji) from quake-affected areas by the student’s t test(*P* > 0.05,respectively). What’s more, the mean increase in IBS-QOL total score from baseline was greater at year 5 in each school in the education group than that in no-education group no matter in quake-unaffected areas or quake-affected areas. (*P* < 0.05,respectively).

### Secondary objectives

**Fig. 4:**
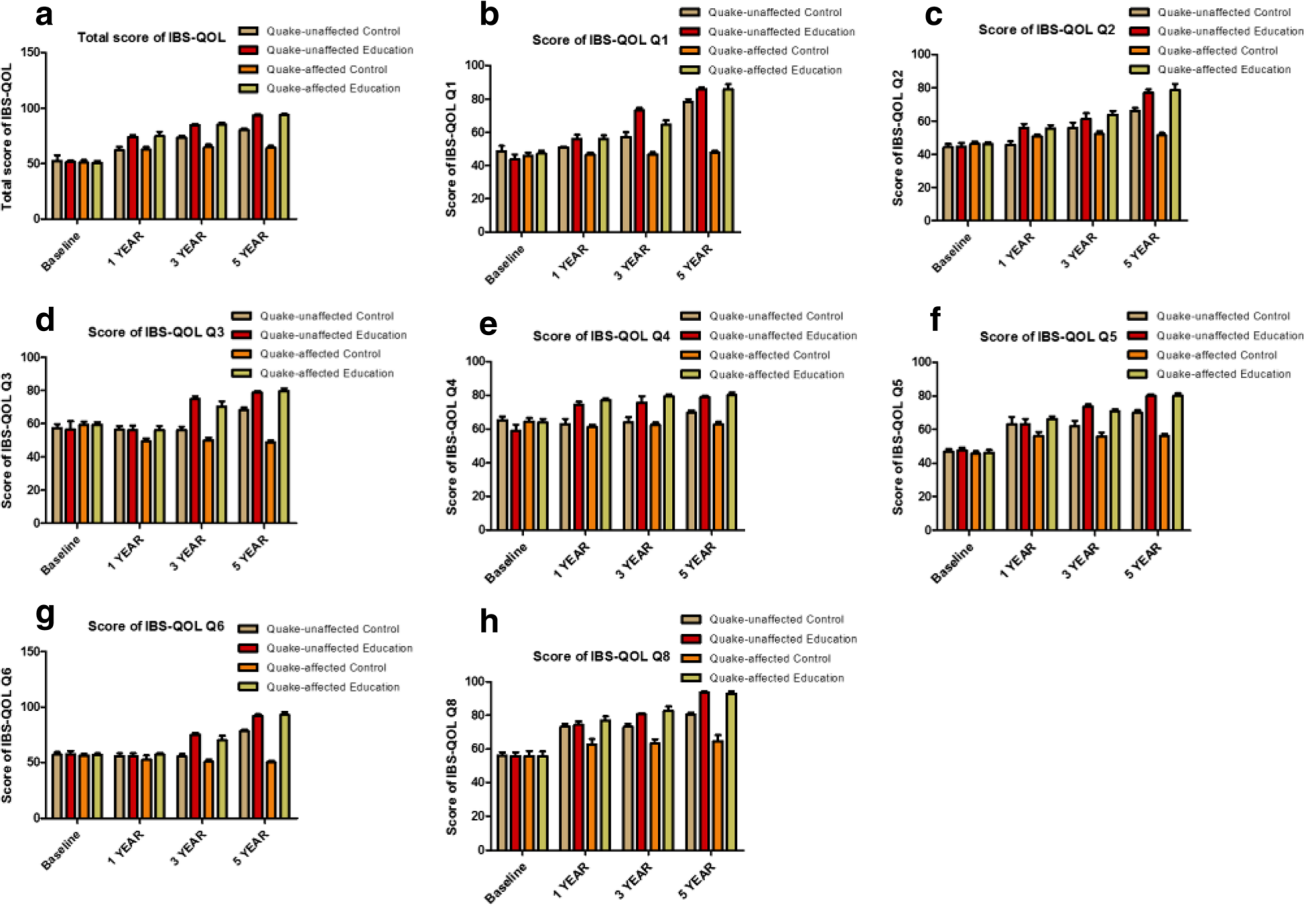
Change of IBS-QOL total score and its subscales’ scores at years 1,3 and 5 in different subgroups, divided by education and areas. **a**: Total score of IBS-QOL; **b**: The score of Dysphoria(Q1); **c**: The score of Interference with activity(Q2); **d**: The score of Body image(Q3); **e**: The score of Health worry(Q4) **f**: The score of Food avoidance(Q5), **g**: The score of Social reaction(Q6); **h**: The score of Relationships(Q8). IBS-QOL, questionnaires of IBS quality of life instrument

Figure 4 shows the scores for the seven IBS-QOL subscales(mean + SD) increased gradually from baseline in each of the subgroups in 5 years(*P* < 0.001). Moreover, the scores of IBS-QOL subscales in the education group were greater than those in the no-education group no matter in quake-unaffected area and in quake-affected areas(*P* < 0.001). Statistically, there was a reciprocal action between factor 1(health education or not) and factor 2(affected by the earthquake or not) at year 1 regarding IBS-QOL for dysphoria (Q1), interference with activity (Q2), body image(Q3), food avoidance (Q5), social reactions (Q6) and relationships (Q8) (*P* < 0.001). Furthermore, the statistical reciprocal action between factor 1(health education or not) and factor 2(affected by the earthquake or not) at year 3 was seen in the IBS-QOL total score, Q1, Q2, Q5 and Q8 (*P* < 0.001). At year 5, the statistical reciprocal action between factor 1(health education or not) and factor 2(affected by the earthquake or not) was noted in the IBS-QOL total score, Q1,Q6 and Q8 (*P* < 0.001).

**Fig. 5:**
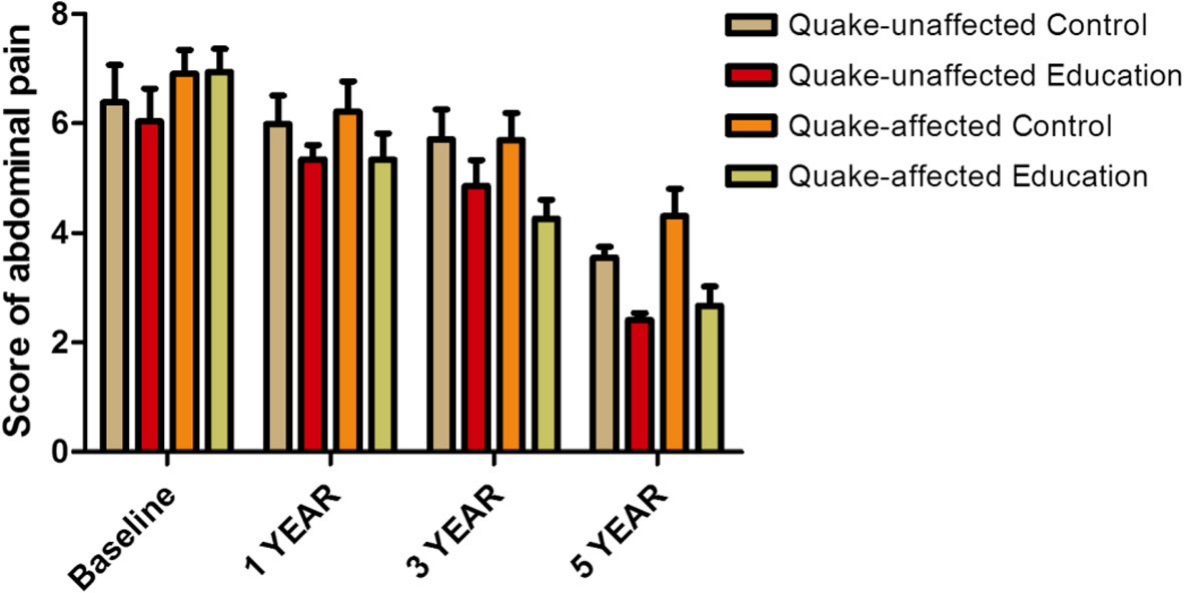
Change of abdominal pain symptom scores at years 1,3 and 5 in different subgroups, divided by education and areas

As shown in Fig. 5, abdominal pain symptom scores gradually reduced from baseline in each subgroup over 5 years (*P* < 0.001). The change in abdominal pain symptom scores in the education group was greater than that in the no-education group no matter in quake-unaffected area and in quake-affected areas(*P* < 0.001). Statistically, there was a reciprocal action between factor 1(health education or not) and factor 2(duration of follow-up) regarding the mean abdominal pain symptom score in both quake-unaffected areas and quake-affected areas (*P* = 0.029 and *P* < 0.001).

## Discussion

Our study was designed to assess the outcome of a 5-year IBS education program in middle school students with IBS in Wenchuan quake-affected areas. It stemmed from our previous study which found that the incidence of anxiety-related emotional disorders among IBS students in Wenchuan quake-affected areas was higher than that in non-IBS students and in students not in quake-affected areas, demonstrating that the stress caused by an earthquake can increase anxiety-related emotional disorders in IBS students [18]. The studies by Cho [19] and Sahoo et al [20] showed that stress events, anxiety and depression were observed in IBS and that they influenced the QOL of patients. Ringström et al [21] revealed that the establishment of an IBS school program could offer information to students with IBS and was a suitable method of treatment. In addition, Susanta Kumar Padhy et al [22] performed an analysis of psychosocial factors which caused predisposition, precipitation, and perpetuation of IBS symptoms. The IBS-QOL questionnaire, which was developed by Patrick and Drossman, showed changes in the quality of life of IBS students after the centralized health education program. However, we deleted the question about sex in order to fit the profile of these middle school students, and increased the VAS evaluation for abdominal pain symptoms to correlate with the IBS-QOL assessment. Thus, two well-validated evaluation methods were adopted in our study.

Our main finding was that the total score of IBS-QOL and the scores of the seven IBS-QOL subscales gradually increased from baseline in each of the subgroups after the 5-year education program, and the abdominal pain symptom score gradually declined. In addition, the variations in IBS-QOL total score, the IBS-QOL subscale scores, and abdominal pain symptom scores in the education group were greater than those in the no-education group. In the first year, there was a clear change in the scores of IBS-QOL for dysphoria, interference with activity, body image, food avoidance, social reactions and relationships with health education in quake-affected areas. In the third year, there was a similar change in IBS-QOL scores for dysphoria, interference with activity, food avoidance and relationships. In year 5, there was also a change in IBS-QOL scores for dysphoria, social reactions and relationships. Our study indicated that health education improved the quality of life and abdominal pain symptoms of IBS students in quake-affected areas, helped to establish good relationships between doctors and IBS students, and eliminated students’ misunderstandings regarding of IBS. These findings are in line with those of Edebol Carlman et al.*..* who also suggested face to face cognitive-behavioral therapy for IBS [9, 23, 24]. However, Hsueh et al emphasized individualized and self-management programs [25], but did not include the treatment of IBS patients who had experienced an earthquake. We also found that identifying and avoiding triggers that could lead to anxiety and dysphoria were important. From the analysis of abdominal pain symptoms, we concluded that the longer the education program, the lower the pain score and improved symptoms [26].These results with IBS of Rome IV diagnosis indexes are mainly composed of the ‘abdominal pain’ in matches [27, 28]. Moreover, the improvement in abdominal pain in IBS students in the quake-affected areas was significantly better than that in the quake-unaffected areas.

The strengths of our study are as follows: this was a five-year multi-center study, and the research subjects affected by the seismic events were chosen using multi-dimensional evaluation results. The weakness of this study is that it started two years after the earthquake. This time gap may have weakened the association between the earthquake, IBS symptoms and the education program.

Our previous study has demonstrated that the stress caused by Wenchuan earthquake on May 12, 2008 can increase anxiety-related emotional disorders in IBS students [18]. In addition, we have found that the health education program significantly improved the quality of life of middle school students with IBS in Wenchuan quake-affected areas. Thus, this study provides us with a new clue to relieve the pains among those IBS students, apart from drug intervention.

## Conclusions

We concluded that the health education program significantly improved the quality of life and abdominal pain of middle school students with IBS in Wenchuan quake-affected areas, with a significant improvement in dysphoria, interference with activity and relationships.

## Abbreviations

CBT: Cognitive behavioral therapy
GSH: Guided self-help
IBS: Irritable bowel syndrome
IBS-C: IBS with constipation
IBS-D: IBS with diarrhoea
IBS-M: IBS with mixed type
IBS-QOL: IBS-specific quality of life
IBS-U: IBS with uncertainty
